# ROS-triggered endothelial cell death mechanisms: Focus on pyroptosis, parthanatos, and ferroptosis

**DOI:** 10.3389/fimmu.2022.1039241

**Published:** 2022-11-01

**Authors:** Dongdong Zheng, Jia Liu, Hulin Piao, Zhicheng Zhu, Ran Wei, Kexiang Liu

**Affiliations:** ^1^Department of Cardiovascular Surgery of the Second Hospital of Jilin University, Changchun, Jilin, China; ^2^Department of Cardiology, The Second Hospital of Jilin University, Changchun, China

**Keywords:** reactive oxygen species, pyroptosis, parthanatos, ferroptosis, endothelial cells

## Abstract

The endothelium is a single layer of epithelium covering the surface of the vascular system, and it represents a physical barrier between the blood and vessel wall that plays an important role in maintaining intravascular homeostasis. However, endothelial dysfunction or endothelial cell death can cause vascular barrier disruption, vasoconstriction and diastolic dysfunction, vascular smooth muscle cell proliferation and migration, inflammatory responses, and thrombosis, which are closely associated with the progression of several diseases, such as atherosclerosis, hypertension, coronary atherosclerotic heart disease, ischemic stroke, acute lung injury, acute kidney injury, diabetic retinopathy, and Alzheimer’s disease. Oxidative stress caused by the overproduction of reactive oxygen species (ROS) is an important mechanism underlying endothelial cell death. Growing evidence suggests that ROS can trigger endothelial cell death in various ways, including pyroptosis, parthanatos, and ferroptosis. Therefore, this review will systematically illustrate the source of ROS in endothelial cells (ECs); reveal the molecular mechanism by which ROS trigger pyroptosis, parthanatos, and ferroptosis in ECs; and provide new ideas for the research and treatment of endothelial dysfunction-related diseases.

## 1 Introduction

**Figure 1:**
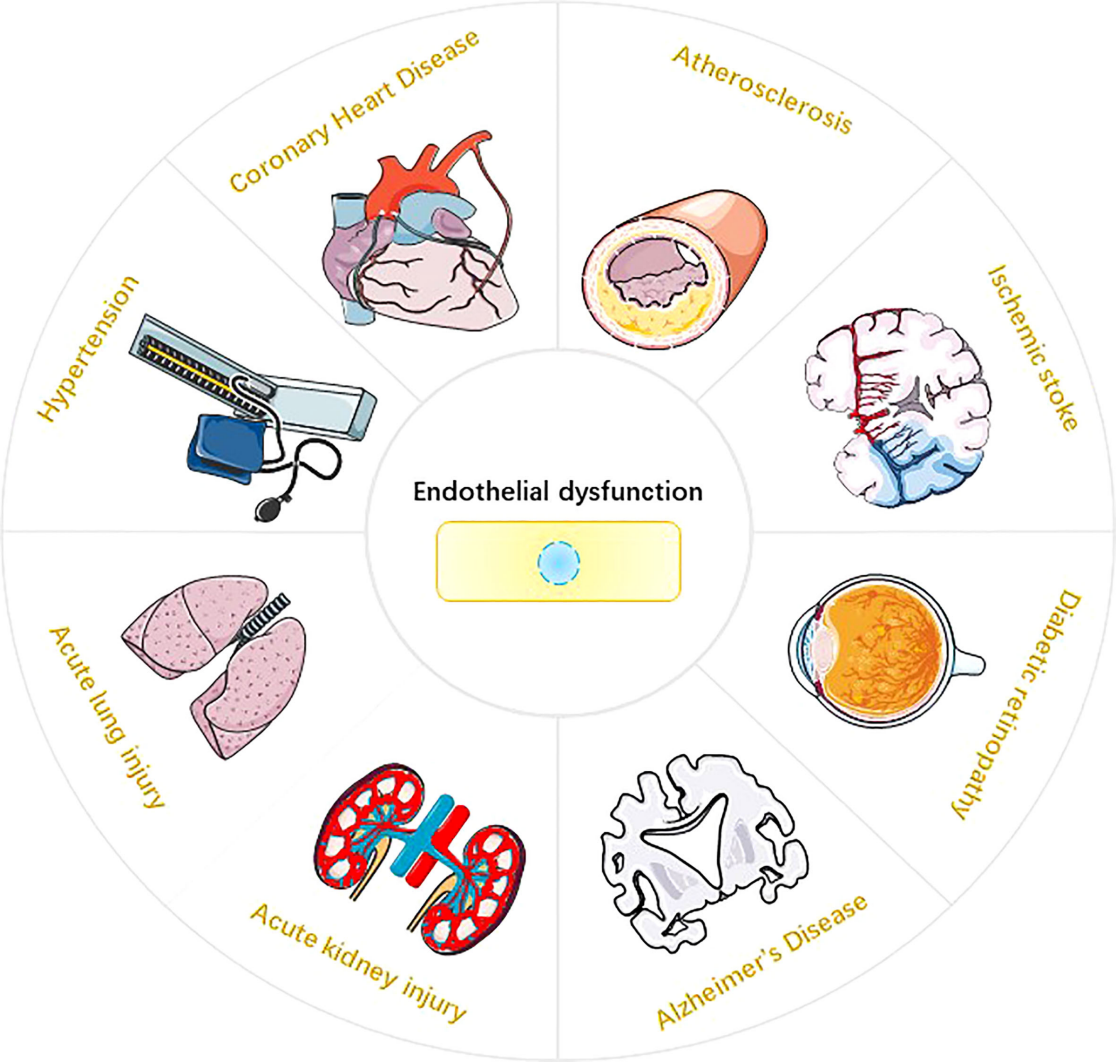
Endothelial dysfunction and Disease. Endothelial dysfunction is involved in the pathophysiological process of various diseases (10-17), such as atherosclerosis, hypertension, coronary atherosclerotic heart disease, ischemic stroke, acute lung injury, acute kidney injury, diabetic retinopathy, and Alzheimer’s disease.

Endothelium is the highly active monolayer of epithelium that covers the surface of blood vessels. Endothelium plays an important role in maintaining vasomotor, coagulation and anticoagulation systems, immune regulation, vascular smooth muscle proliferation and migration (1–3). Reactive oxygen species (ROS) in endothelial cells (EC) are mainly derived from mitochondria, NADPH oxidase (NOXs), eNOS uncoupling and xanthine oxidase (XO) (4, 5). Under physiological conditions, ROS are essential for physiological cellular functions such as host defense, post-translational processing of proteins, cell signaling, regulation of gene expression, and cell differentiation (6). However, ROS overproduction may cause endothelial dysfunction (ED) and endothelial cell death. The impairment of NO synthesis marks the onset of ED, which is mainly mediated by the eNOS uncoupling mechanism (7). In the process of ROS-mediated ED, the expression of various pro-inflammatory cytokines, i.e., interleukin-1β (interleukin-1β), interleukin-18 (interluekin-18, IL-18), and cell adhesion molecules, i.e., intercellular adhesion molecule-1 (ICAM-1), vascular cell adhesion molecule-1 (VCAM-1), and E-selectin may be promoted in endothelial cells. These molecules are closely related to the occurrence of inflammatory responses (8, 9). In addition, ROS can mediate a variety of programmed cell death (PCD) in endothelial cells, such as pyroptosis, parthanatos and ferroptosisis. It is worth noting that endothelial dysfunction or endothelial cell death is closely related to the occurrence and development of various diseases, such as atherosclerosis (10), coronary heart disease (11), hypertension (12), ischemic stroke (13), acute lung injury (14), acute kidney injury (15), diabetic retinopathy (16) and Alzheimer’s disease (17) (**Figure 1**). This review systematically elucidates the sources of ROS in EC; covers the molecular mechanisms of ROS-induced pyroptosis, parthanatos and ferroptosis in EC cells; and provide new insights for the research and treatment of endothelial cell death-related diseases.

## 2 Sources of ROS in ECs

**Figure 2:**
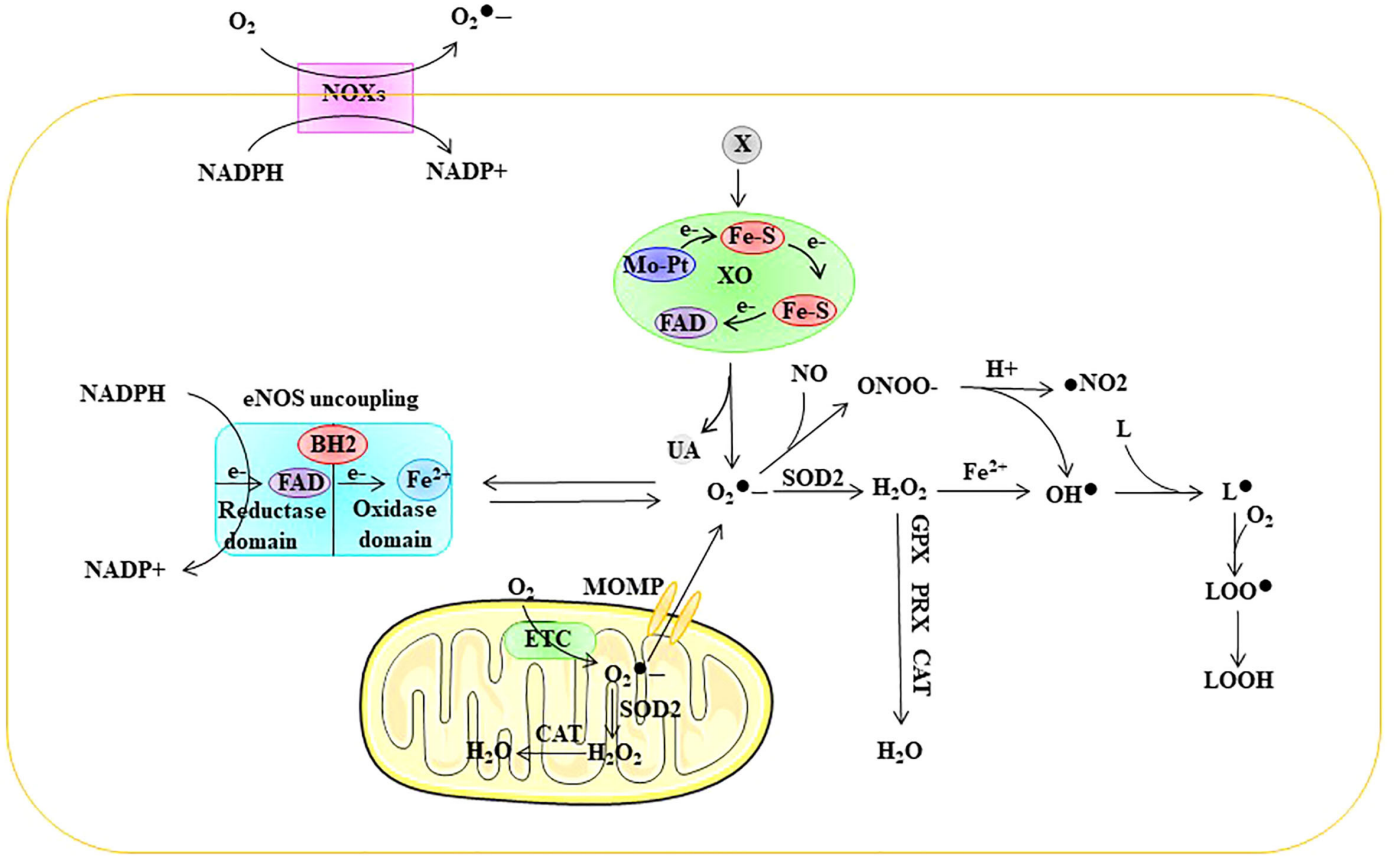
Sources of ROS in ECs. ROS in ECs are mainly derived from mitochondria, NOXs, eNOS uncoupling, and XO. ETC electron transport chain, eNOS endothelial nitric oxide synthase, FAD flavin adenine dinuc-leotide, Fe-S iron-sulfur center, Mo-co molybdenum cofactor, MOMP mi-tochondrial outer membrane permeabilization, NOX NADPH oxidase, SOD superoxide dismutase, GPX glutathione peroxidase, PRX peroxired-oxin, CAT catalase, ${\text{O}}_{2}^{•–}$ superoxide, ONOO^•^ peroxynitrite anion, H_2_O_2_ hydrogen peroxide, OH^•^ peroxyl radical, L lipid, L^•^ lipid free radical, LOO^•^ lipid peroxy radical, LOOH lipid peroxide, UA uric acid, X xanthine, XO xanthine oxidase.

Intracellular ROS are mainly composed of superoxide anions ( ${\text{O}}_{2}^{•–}$ ), hydrogen peroxide (H_2_O_2_), and hydroxyl radicals (OH^•^) (18). O_2_ forms ${\text{O}}_{2}^{•–}$ by capturing an electron, which leads to the generation of other ROS. ${\text{O}}_{2}^{•–}$ is unstable in aqueous solutions due to its short half-life; therefore, intracellular ${\text{O}}_{2}^{•–}$ is quickly scavenged or converted to other forms of ROS. ${\text{O}}_{2}^{•–}$ is cleared or converted mainly *via* three pathways:1) ${\text{O}}_{2}^{•–}$ generates H_2_O_2_ through the action of superoxide dismutase (SOD); 2) low concentrations (picomolar range) of ${\text{O}}_{2}^{•–}$ interact with nitric oxide (NO) to generate peroxynitrite anion (ONOO^•^), which occurs even faster than disproportionation to generate H_2_O_2_; and 3) high concentrations of ${\text{O}}_{2}^{•–}$ generate OH^•^ through the Fenton reaction with H_2_O_2_ (18). In addition, OH^•^ reacts with fatty acids to generate lipid free radicals (L^•^). ROS in ECs are mainly derived from mitochondria, NADPH oxidase (NOX), endothelial NOS (eNOS) uncoupling, and xanthine oxidase (XO) (4, 5) (**Figure 2**).

### 2.1 Mitochondria

Mitochondria are the source of cellular power and produce ATP through oxidative phosphorylation (OXPHO), which accounts for approximately 80% of the energy requirements, with glycolysis accounting for the remaining 20%. Mitochondrial ROS production results from oxidative phosphorylation associated with aerobic respiration within the mitochondrial electron transport chain (ETC). Mitochondrial complexes I and III are the major sites for the generation of ${\text{O}}_{2}^{•–}$ (19–21). Electron leakage from the ETC results in the reduction of O_2_ to ${\text{O}}_{2}^{•–}$ rather than to H_2_O. SOD further disproportionates mitochondrial ${\text{O}}_{2}^{•–}$ to form H_2_O_2_. Approximately 1-2% of O_2_ entering the ETC is estimated to be converted into ROS (22) (**Figure 2**). Moreover, mitochondrial ROS overproduction is one of the causes of EC dysfunction. For example, Rao et al. showed that nicotinamide nucleotide transhydrogenase (NNT) knockout resulted in a significant increase in mitochondrial ROS production and glutathione peroxidase activity and a decline in glutathione reductase activity (23).

### 2.2 NAPDH oxidase

#### 2.2.1 Structure

NAPDH oxidase (NOX) is an important source of ROS in cells. The NOX family includes NOX1, NOX2, NOX3, NOX4, NOX5, and dual oxidases (DUOX1 and DUOX2) (22). NOXs are multi-transmembrane proteins whose C-termini are exposed in the cytoplasm, and they share common domains, including six conserved transmembrane domains, four conserved heme-binding histidines, flavin adenine dinucleotide (FAD)-binding domains, and NADPH-binding domains (24). NOX in turn transfers electrons from NADPH to FAD, the heme group, and then to O_2_, resulting in ${\text{O}}_{2}^{•–}$ and/or H_2_O_2_ production (25).

#### 2.2.2 NOXs activation in ED

**Figure 3:**
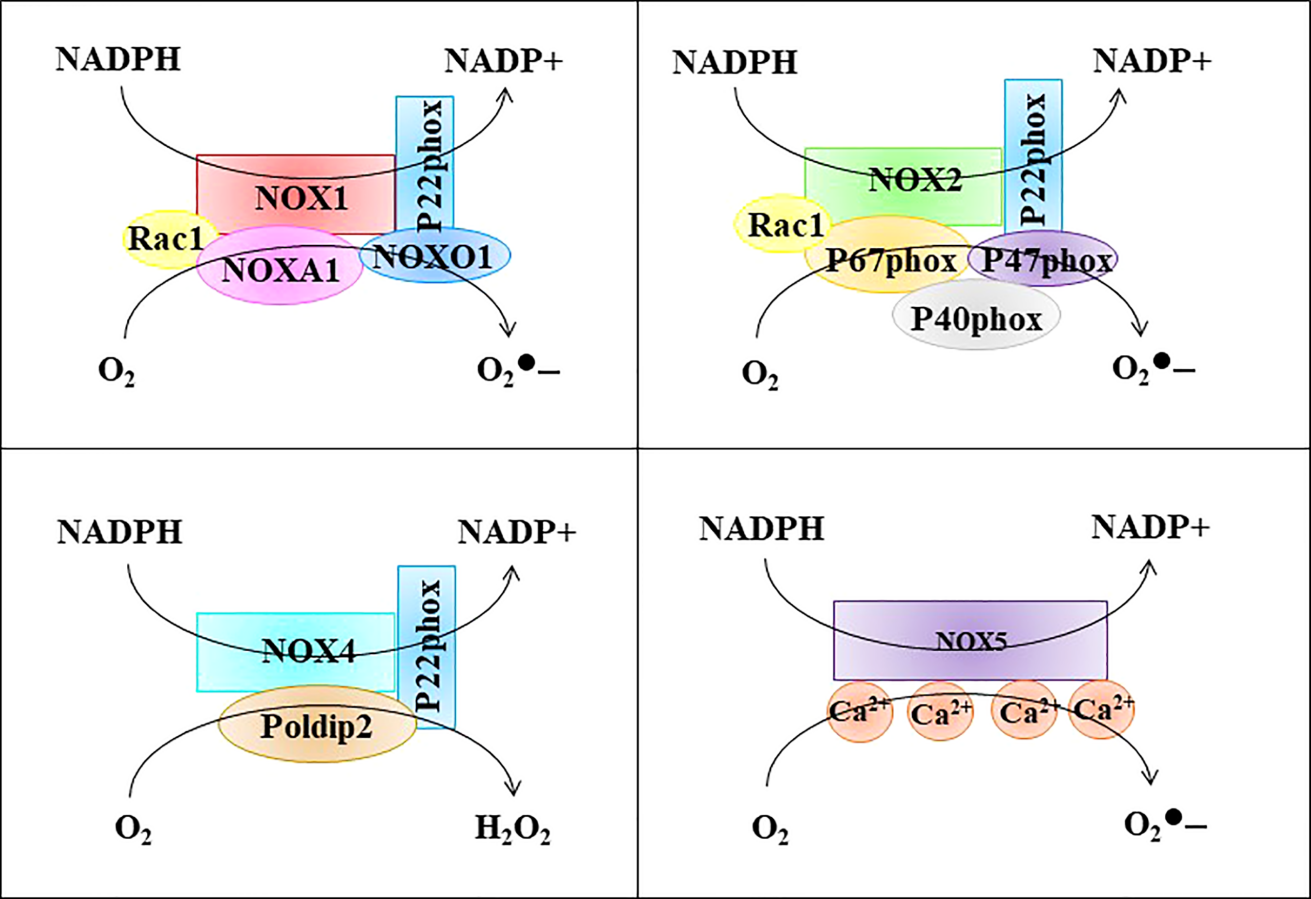
The structure of NOXs. The main subtypes of NOX in ECs include NOX1, NOX2, NOX4, and NOX5. The catalytic product of NOX1, NOX2, and NOX5 is ${\text{O}}_{2}^{•–}$ , while the catalytic product of NOX4 is H_2_O_2_.

The main subtypes of NOX in ECs include NOX1, NOX2, NOX4, and NOX5 (25, 26). The catalytic product of NOX1, NOX2, and NOX5 is ${\text{O}}_{2}^{•–}$ , while the catalytic product of NOX4 is H_2_O_2_ (**Figure 3**). NOX complexes consist of catalytic subunits (NOX) and regulatory subunits, with the exception of NOX5, which consists of only one catalytic subunit (22). NOX2 is the first NOX isoform identified in ECs and represents the most widely and deeply studied isoform; therefore, we first discuss its activation mechanism. Under resting conditions, NOX2 and p22phox are located on the membrane as inactive complexes while p40phox, p67phox, and p47phox subunits are located in the cytoplasm (22). Activation of NOX2 also requires the small GTPase Rac1. Activation of Rac1 initiates NOX2, and Rac1 is recruited to the membrane and then recruits other cytosolic components (27). p47phox is then phosphorylated by protein kinase C (PKC) and transferred to the membrane together with p67phox and p40phox (28). Next, the phosphorylation of p47phox can combine with p22phox to realize the assembly and activation of the NOX2 complex (29). The basal activity of NOX2 in ECs is low, although it is rapidly activated by pathological causative factors, such as hyperlipidemia, hypertension, and hyperglycemia (30). EC injury in the early stages of vascular disease has been reported to be mediated by excess NOX2-derived superoxide (31). Similar to NOX2, NOX1 activation requires the assembly of multiple subunits. During NOX1 activation, the activation function of p67phox is performed by NOXA1 and the organizer function of p47phox is performed by NOXO1 (32, 33). Compared with p47phox, NOXO1 does not contain an auto-inhibitor domain; therefore, the NOX1-NOXO1-NOXA1 complex has high basal activity (29). Reports have indicated that endothelin-1 (ET-1) overexpression in ECs promotes atherosclerosis progression through NOX1 in type 1 diabetes, perivascular oxidative stress, and inflammation (34). Furthermore, NOX1 is involved in eNOS uncoupling in ECs. For example, Youn et al. found that NOX1 activation in streptozotocin-induced diabetic mice is dependent on p47phox and NOXO1 and mediates eNOS uncoupling. NOX1 knockout mice are protected from ED (35). NOX4 is the most highly expressed NOX homolog in ECs. Compared to NOX1 and NOX2, activation of NOX4 requires only p22phox and polymerase delta-interacting protein 2 (Poldip2) (30, 36). Several studies have suggested that NOX4 plays an important role in ED. For example, Jiang et al. found that NOX4 knockdown attenuated pulmonary ROS production in septic mice, attenuated redox-sensitive activation of the CaMKII/ERK1/2/MLCK pathway, and restored the expression of the tight junction proteins ZO-1 and occludin to maintain the integrity of the EC barrier (37). Zhao et al. showed that tert-butyl hydroperoxide (t-BHP) induces EC apoptosis through NOX4 (38). However, there are also reports that NOX4 protects ECs during oxidative stress. This may be related to the generation of H_2_O_2_ by NOX4. H_2_O_2_ is considered an important signaling intermediate because of its ability to selectively and reversibly oxidize reactive cysteine residues, thereby altering the function of protein targets including phosphatases, kinases, ion channels, and transcription factors (39). In EC, these effects ultimately lead to increased expression and activity of important angioprotective enzymes, including eNOS (40). Furthermore, unlike superoxide, H_2_O_2_ does not react appreciably with NO, and thus does not reduce NO bioavailability (39). Unlike NOX1, NOX2, and NOX4, the activation of NOX5 does not depend on other subunits. NOX5 contains an N-terminal calmodulin-like domain with four Ca2+ binding sites (EF hands) (39). Therefore, NOX5 activity can be directly regulated by changes in the intracellular [Ca2+]. Evidence suggests that NOX5 plays an important role in ED. Silva et al. found that lysophosphatidylcholine drives NOX5-dependent ROS production in ECs *via* calcium influx, leading to ED (40). Elbatreek et al. found that NOX5 overexpression in mice caused eNOS uncoupling, thus leading to ED (41). Therefore, ROS derived from NOXs play an important role in mediating ED.

#### 2.3 eNOS uncoupling

Nitric oxide (NO) plays an important role in maintaining vascular homeostasis owing to its vasodilatory effects. Nitric oxide synthase (NOS) is synthesized from l-arginine and O_2_ and represents a key enzyme involved in nitric oxide (NO) synthesis. There are three subtypes of NOS: neuronal NOS (nNOS), inducible NOS (iNOS), and endothelial NOS (eNOS) (42). NOS functions as a homodimer during NO biosynthesis. Each monomer has an oxygenase domain at the N-terminus and a reductase domain at the C-terminus. The oxygenase domain consists of binding sites for FAD, FMN, and NADPH and is linked to the reductase domain through a calmodulin recognition site. The reductase domain contains binding sites for heme, tetrahydrobiopterin (BH4), and l-arginine. The formation of NO requires electron flow, which starts at the flavin level in the reductase domain and ends at the heme level in the oxygenase domain (7). Specifically, NADPH releases electrons in the reductase domain and transfers them to heme *via* FAD and FMN. In the presence of l-arginine and cofactor BH4, electrons can reduce O_2_ to form NO and l-citrulline (43, 44). The presence of BH4 is critical for NO formation because it is involved in l-arginine binding and electron transfer. During ED, BH4 depletion is considered the main mechanism by which eNOS uncoupling generates ROS (45, 46). In fact, in the absence of BH4, l-arginine cannot bind to its site and the terminal electron acceptor becomes O_2_, thus forming ${\text{O}}_{2}^{•–}$ instead of NO, a process defined as eNOS uncoupling (47, 48). Notably, ROS derived from the NOX system are closely related to the depletion of BH4 (30). Furthermore, ${\text{O}}_{2}^{•–}$ reacts with NO to form ONOO^•^, which can lead to the oxidation of iron-sulfur centers and eNOS core ZnS4 (4, 49). Taken together, these results suggest that eNOS decoupling is closely associated with ED (**Figure 2**).

### 2.4 Xanthine oxidase

Xanthine oxidoreductase (XOR) exists in two different forms, xanthine dehydrogenase (XDH) and XO, and they represent the rate-limiting enzymes in purine metabolism (50). Normally, XOR exists in the cells in the form of XDH. XDH is a homodimer of approximately 300 kDa, with four redox centers in each subunit: a molybdenum cofactor (Mo-co), two iron-sulfur (Fe-S) centers, and a flavin adenine dinucleotide (FAD) domain (51). XDH catalyzes the oxidation of hypoxanthine to xanthine and xanthine to uric acid at the Mo-co site, and electrons shuttle through two Fe-S centers to the FAD binding site, where NAD+ is reduced to NADH (51). Under physiological conditions, XOR is mainly present in ECs in the form of XDH (52). XDH can break down hypoxanthine into uric acid (53) and reduce nitrite to produce NO, which helps regulate vasodilation and blood pressure (53). However, under oxidative stress conditions, ROS can oxidize cystine thiols on XDH, resulting in the conversion of XDH to XO (30, 54). The main difference between XO and XDH is their oxidative substrate affinity, where XO has a reduced affinity for NAD+ and more than 11-fold increased affinity for O_2_ (54). While promoting the decomposition of hypoxanthine into uric acid, XO generates ${\text{O}}_{2}^{•–}$ through one-electron reduction, and H_2_O_2_ through two-electron reduction (55). Reports have indicated that XO-induced ED is closely related to its by-products, including ROS and uric acid (56, 57). Intracellular uric acid can exacerbate oxidative stress in ECs, thereby causing ED (52). Therefore, XO is an important source of ROS in ECs and closely related to ED (**Figure 2**).

## 3 Pyroptosis

Pyroptosis is a type of programmed cell death caused by various stimuli. The molecular features of pyroptosis include inflammasome assembly and activation, membrane pore formation, and pro-inflammatory cytokine maturation and release. Depending on whether pyroptosis requires caspase-1 activation, it can be divided into the classical and non-classical inflammasome pathways.

### 3.1 Inflammasome

**Figure 4:**
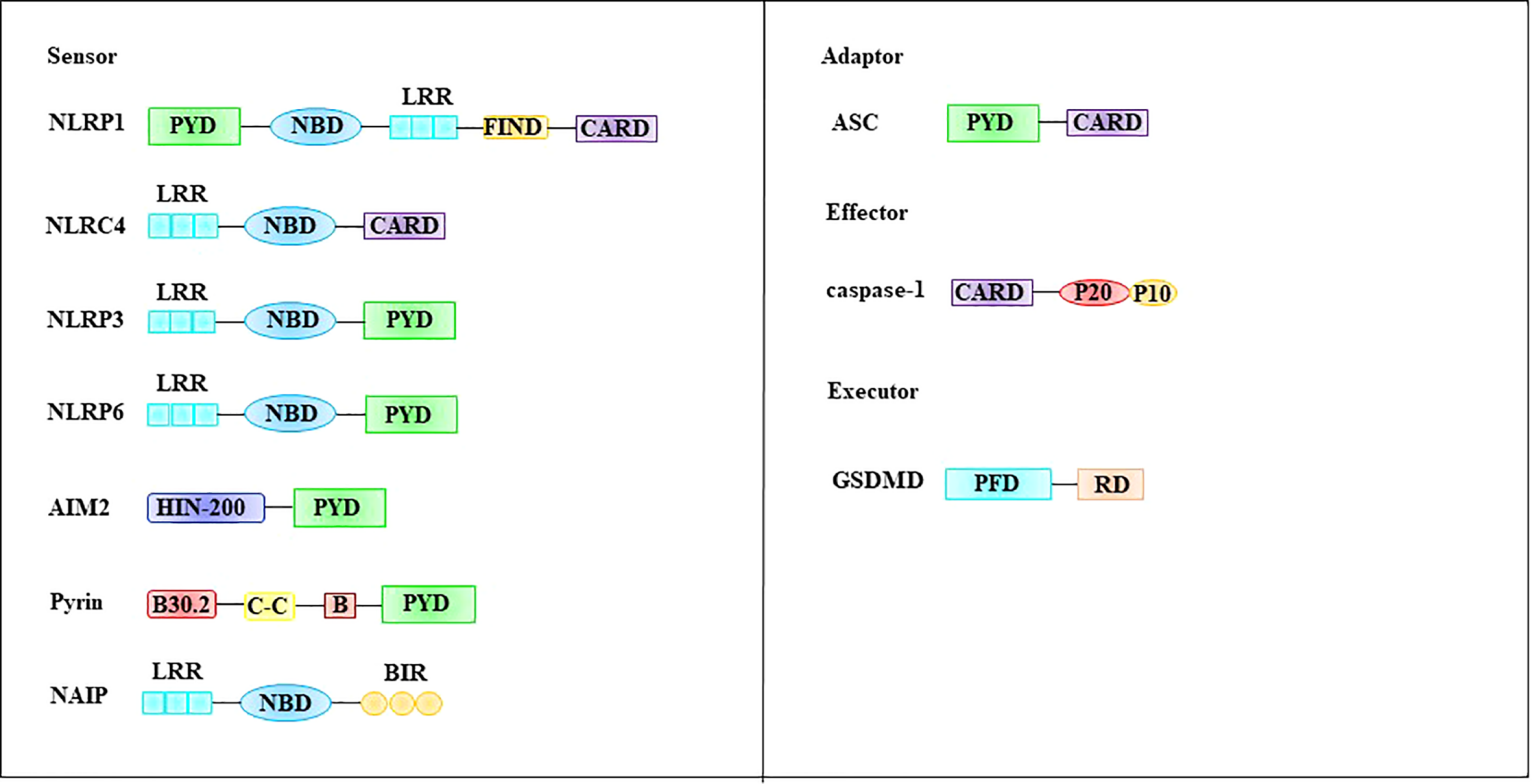
Molecular structures involved in pyroptosis. The inflammasome is composed of the intracellular recognition receptor, adaptor protein ASC, and effector protein caspase-1. GSDMD, a family of pore-forming effector proteins, is thought to be the executor of pyroptosis. ASC apoptosis-associated speck-like protein, CARD, caspase-recruitment domain, FIIND function-to-find domain, GSDMD Gasdermin D, LRR leucine-rich repeat domain, NBD nucleotide-binding domain, PFD pore-forming domain, PYD pyrin domain, RD repressor domain.

The inflammasome is composed of the intracellular recognition receptor, adaptor protein apoptosis-associated speck-like protein (ASC), and effector protein caspase-1 (57). The intracellular recognition receptors that constitute the inflammasome include the NOD-like receptor (NLR) protein family of AIM2-like receptors (ALRs) and pyrin, which can directly or indirectly activate ASC to activate caspase-1 (58, 59). Structurally, these intracellular recognition receptors contain a CARD or PYD domain at their N-terminus. ASC contains a PYD structure and a CARD domain, and caspase-1 contains a CARD domain (60). The intracellular recognition receptors NLRP1, NLRP3, NLRP6, AIM2, and pyrin all contain PYD at their N-termini, whereas NLRP1 and NLRC4 contain CARD (57, 61) (**Figure 4**).

### 3.2 Gasdermin D: A mechanism of cell swelling in pyroptosis

Gasdermin D (GSDMD), a family of pore-forming effector proteins, is thought to be the executor of pyroptosis. GSDMD is a member of the gasdermin protein family, which includes GSDMA, GSDMB, GSDMC, GSDMD, GSDME (also known as DFNA5), and PJVK (also known as DFNB59). Most members of this family have been shown to exhibit pore-punching effects (62). Among these, the most extensive and in-depth research has been performed on GSDMD. GSDMD is composed of a pore-forming domain (PFD), linker, and repressor domain (RD) (62, 63) (**Figure 4**). The PFD (also known as N-GSDMD) is located at the N-terminus and consists of 242 amino acids. This part is an important structure for the GSDMD to perform the punching function. RD (also known as C-GSDMD) is located at the C-terminus and consists of 199 amino acids, which is an important structure for inhibiting GSDMD function (63). The linker between PFD and RD is composed of 43 amino acids, and this part is the switch for GSDMD activation (63). During pyroptosis, the linker of GSDMD can be cleaved by activated caspase-1 or caspase-4/5/11, and C-GSDMD dissociates from GSDMD, releasing its inhibitory effect on N-GSDMD (64, 65). Subsequently, N-GSDMD was integrated into the cell membrane, and approximately 16 PFD monomers were oligomerized to form membrane pores with a diameter of 10-15 nm. The formation of membrane pores causes a loss of cell membrane integrity and breaks the osmotic pressure barrier of the plasma membrane (62, 66). Under normal circumstances, intracellular sodium ions are low and potassium ions are high. However, extracellular fluid is high in sodium ions and low in potassium ions. The formation of this intracellular and extracellular ion concentration difference is dependent on the Na^+^ pump (Na^+^-K^+^-ATPase). Na^+^-K^+^-ATPase is widely expressed on the cell membrane surface and acts as a sodium-potassium antiporter. Each Na^+^-K^+^-ATPase can transport three sodium ions from the intracellular to extracellular space and two potassium ions into the cell by consuming one molecule of ATP (67). This asymmetric cation transport mechanism plays an important role in maintaining differences in the chemical concentration gradients of sodium and potassium ions inside and outside the cell. Notably, this asymmetric cation transport mechanism mediates cell swelling together with N-GSDMD. Specifically, when N-GSDMD forms pores in the cell membrane, the force generated by the concentration gradient expelling potassium ions out of the cell is roughly offset by the electric field force that pulls potassium ions into the cytoplasm, resulting in the passage of potassium ions through the membrane pores. Therefore, the flux is minimized. In contrast, both the sodium ion concentration gradient and electric field force promote the entry of sodium ions into cells, resulting in a large influx of sodium ions (62). The influx of sodium ions is accompanied by the entry of water molecules, which causes cells to swell or even rupture.

Physiologically, interleukin-1β (IL-1β) and interleukin-18 (IL-18) exist in inactive precursor forms, namely pro-IL-1β and pro-IL-18 (62). However, during pyroptosis, activated caspase-1 cleaves pro-IL-1β and pro-IL-18 to produce mature IL-1β and IL-18 (53). Unlike other cytokines, mature IL-1β and IL-18 are not secreted out of cells *via* the endoplasmic reticulum-Golgi pathway; rather, this action depends on N-GSDMD (68, 69). Therefore, N-GSDMD is an important channel for the secretion of mature IL-1β and IL-18 into the extracellular space during pyroptosis.

### 3.3 Pyroptosis pathway

**Figure 5:**
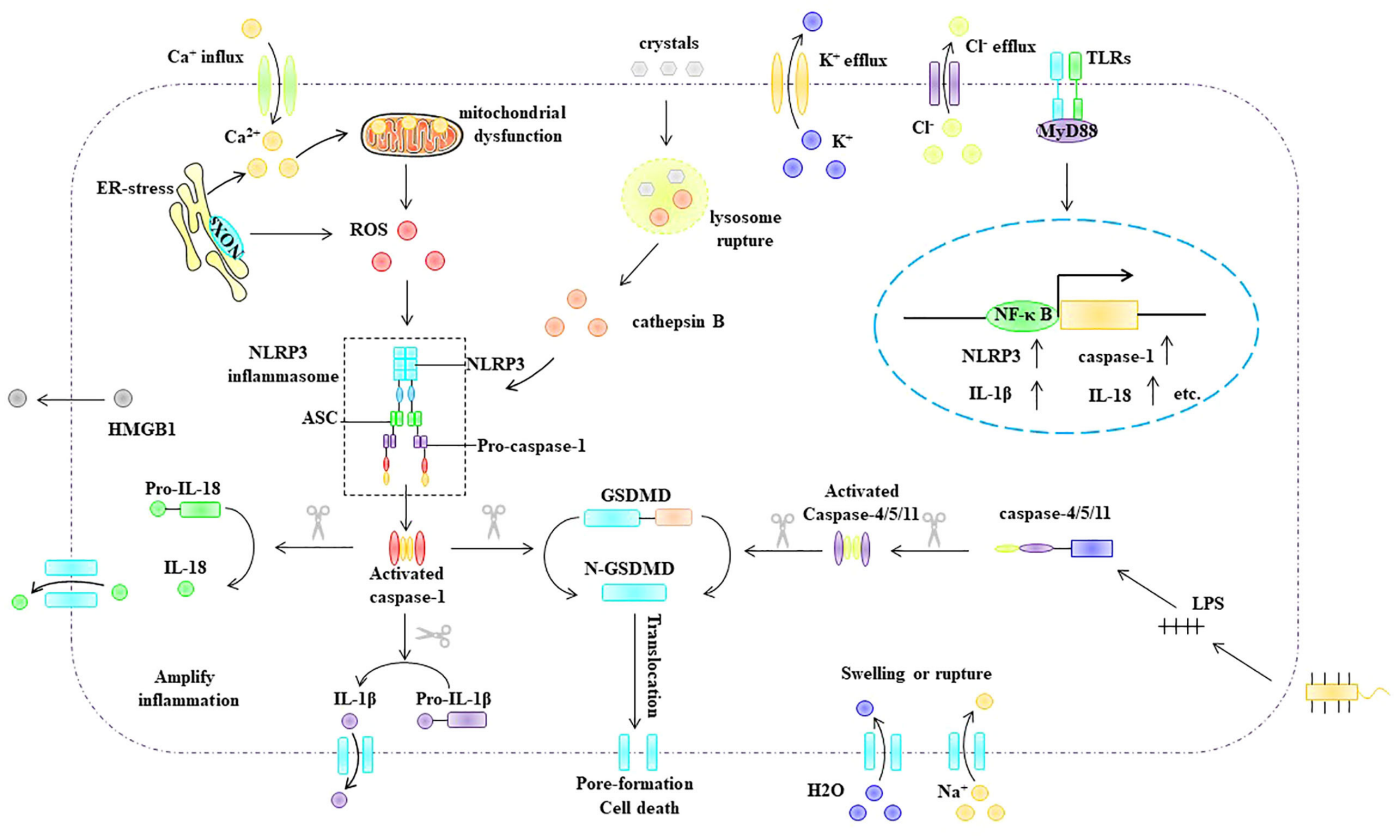
Pyroptosis pathway. Depending on whether pyroptosis requires caspase-1 activation, it can be divided into the classical and non-classical inflammasome pathways. ASC, apoptosis-associated speck-like protein, ER endoplasmic reticulum, GSDMD Gasdermin D, HMGB1 high mobility group box 1, IL-1β interleukin-1β, IL -18 interleukin- 18, MyD88 myeloid differentiation primary response gene 88, NLRP3 NLR-family pyrin domain-containing protein 3NF-κB nuclear factor kappa B, NLRP3 NLR-family pyrin domain-containing protein 3, ox-LDL oxidized low density lipoprotein, ROS reactive oxygen species, TLR Toll-like receptors.

Depending on whether pyroptosis requires caspase-1 activation, it can be divided into the classical and non-classical inflammasome pathways (**Figure 5**). The classical inflammasome pathway mainly includes the assembly and activation of inflammasomes, formation of porins, and maturation and secretion of IL-1β and IL-18. Specifically, intracellular and extracellular PAMPs or DAMPs (e.g., viral dsDNA, bacterial lipopolysaccharide, extracellular ATP, ox-LDL, and cholesterol crystals) can promote inflammasome assembly and activation. Inflammasomes activate pro-caspase-1 *via* self-cleavage. Activated caspase-1 cleaves the porin GSDMD to generate mature N-GSDMD (70) and cleaves pro-IL-1β and pro-IL-18 to generate mature IL-1β and IL-18 (70). Compared with the classical inflammasome pathway, activation of the non-canonical inflammasome pathway does not require the assembly and activation of the inflammasome. The bacterial cell wall component lipopolysaccharide can activate caspase-11 (human) or caspase-4/5 (murine) (71, 72). Activated caspase-4/5/11 cleaves GSDMD to generate mature N-GSDMD (64, 65). Subsequently, N-GSDMD is integrated into the cell membrane to form membrane pores that mediate pyroptosis.

## 4 ROS trigger pyroptosis in EC

Among the classical inflammasome pathways, NLRP3 inflammasome-mediated pyroptosis is the most extensively studied. The NLRP3 inflammasome is composed of the intracellular sensor protein NLRP3, adaptor protein ASC, and effector protein pro-caspase-1 (70, 73). NLRP3 inflammasome activation is thought to include multiple upstream signals, most of which are not mutually exclusive, including potassium (K+) efflux (74, 75), calcium flux (76), endoplasmic reticulum stress (77), mitochondrial dysfunction (78), ROS (79), and lysosomal disruption (80). Notably, ROS, as upstream signals of NLRP3 inflammasome activation, play an important role in NLRP3 inflammasome activation (81–83). Overall, the mechanism by which ROS activate the NLRP3 inflammasome involves two important processes: the initiation phase and the activation phase. The initiation signal indicates that ROS can upregulate the expression of NLRP3, pro-caspase-1, and pro-IL-1β (79). During the activation stage, ROS can promote the assembly and activation of the NLRP3 inflammasome, and thioredoxin-interacting protein (TXNIP) plays an important role in this process. TXNIP has been identified as a reduced thioredoxin protein (Trx) binding protein. When cells are in a quiescent state, TXNIP interacts with the redox domain of Trx and is considered a negative regulator of Trx. However, when intracellular ROS are increased, Trx is oxidized, thus leading to the dissociation of TXNIP from Trx, which subsequently interacts with NLRP3, leading to the assembly and activation of the NLRP3 inflammasome (84, 85).

Numerous recent studies have shown that NLRP3 inflammasome activation plays an important role in mediating ED (61). Increasing evidence has shown that certain stimuli, such as oxidized low-density lipoprotein, hyperglycemia, and nicotine, can activate the NLRP3 inflammasome in EC, thus leading to endothelial cell death. For example, Wu et al. found that ox-LDL induced the upregulation of NLRP3, caspase-1, and IL-1β in ECs in a dose-dependent manner (86). Hang et al. found that ox-LDL stimulated NLRP3 inflammasome activation, increased IL-1β and IL-18 maturation and secretion, increased intracellular ROS, and increased lactate dehydrogenase (LDH) release in ECs (87). Chen et al. found that ox-LDL could induce increases in ROS and upregulate the expression of ICAM-1, TXNIP, NLRP3, and caspase-1 in ECs (88). Zhuang et al. found that forkhead box P transcription factor 1 (Foxp1) is a negative regulator of NLRP3 inflammasome activation in ECs, and they also revealed found that Foxp1 is significantly downregulated in atherosclerosis-susceptible endothelium and Foxp1 knockout in ApoE-/- mice exacerbates atherosclerosis. Subsequently, NLRP3, caspase-1, and pro-IL-1β were significantly upregulated and IL-1β secretion was increased. The team further demonstrated that Foxp1 is a gatekeeper of vascular inflammation and a transcriptional repressor; moreover, it can inhibit the expression of NLRP3, caspase-1, and pro-IL-1β from the transcription initiation level (60). Numerous studies have shown that NOX4 plays an important role in mediating endothelial dysfunction in type 2 diabetes (89). For example, Liao et al. performed *in vitro* and *in vivo* experiments and found that high glucose levels can promote the generation of ROS in ECs by upregulating NOX4 (90). Li et al. found that high levels can upregulate the expression of NOX4, NLRP3, and caspase-1 in ECs and showed that high glucose-induced NLRP3 inflammasome activation was dependent on NOX4 and mediates EC tight junction barrier disruption (91). Dunn et al. found that high glucose levels can upregulate TXNIP expression to induce endothelial dysfunction. In addition, the team found that knockdown of TXNIP could alleviate high glucose-mediated endothelial dysfunction (92). Chen et al. found that silencing NLRP3 could reverse the high glucose-induced upregulation of NLRP3, caspase-1, IL-1β, IL-18, and ICAM-1, and they further found that ROS scavengers could reverse the high glucose-induced upregulation of IL-1β and IL-18 in ECs. Furthermore, the team found that TXNIP knockdown inhibited IL-1β and IL-18 maturation (8). Wu et al. found that nicotine could induce upregulation of NLRP3, caspase-1, ASC, IL-1β, and IL-18 expression in ECs, DNA damage, and LDH release, and they also found that N-acetylcysteine (NAC) could inhibit nicotine-induced inflammasome activation and alleviate DNA damage, indicating that nicotine mediates NLRP3 inflammasome activation in ECs through ROS. The team further found that the knockdown of NLRP3 or ASC could inhibit nicotine-induced activation of the NLRP3 inflammasome in ECs. Similarly, the caspase-1 inhibitor VP-765 also inhibits nicotine-induced activation of the NLRP3 inflammasome in ECs (93). Zhang et al. found that nicotine could activate the NLRP3 inflammasome in EC. The team further found that NLRP3 inflammasome activation can promote the destruction of tight junction proteins between ECs, resulting in increased vascular permeability (94). Cau et al. found that Ang-II induces endothelial dysfunction, vascular remodeling, and hypertension through NLRP3 inflammasome activation (95).

**Figure 6:**
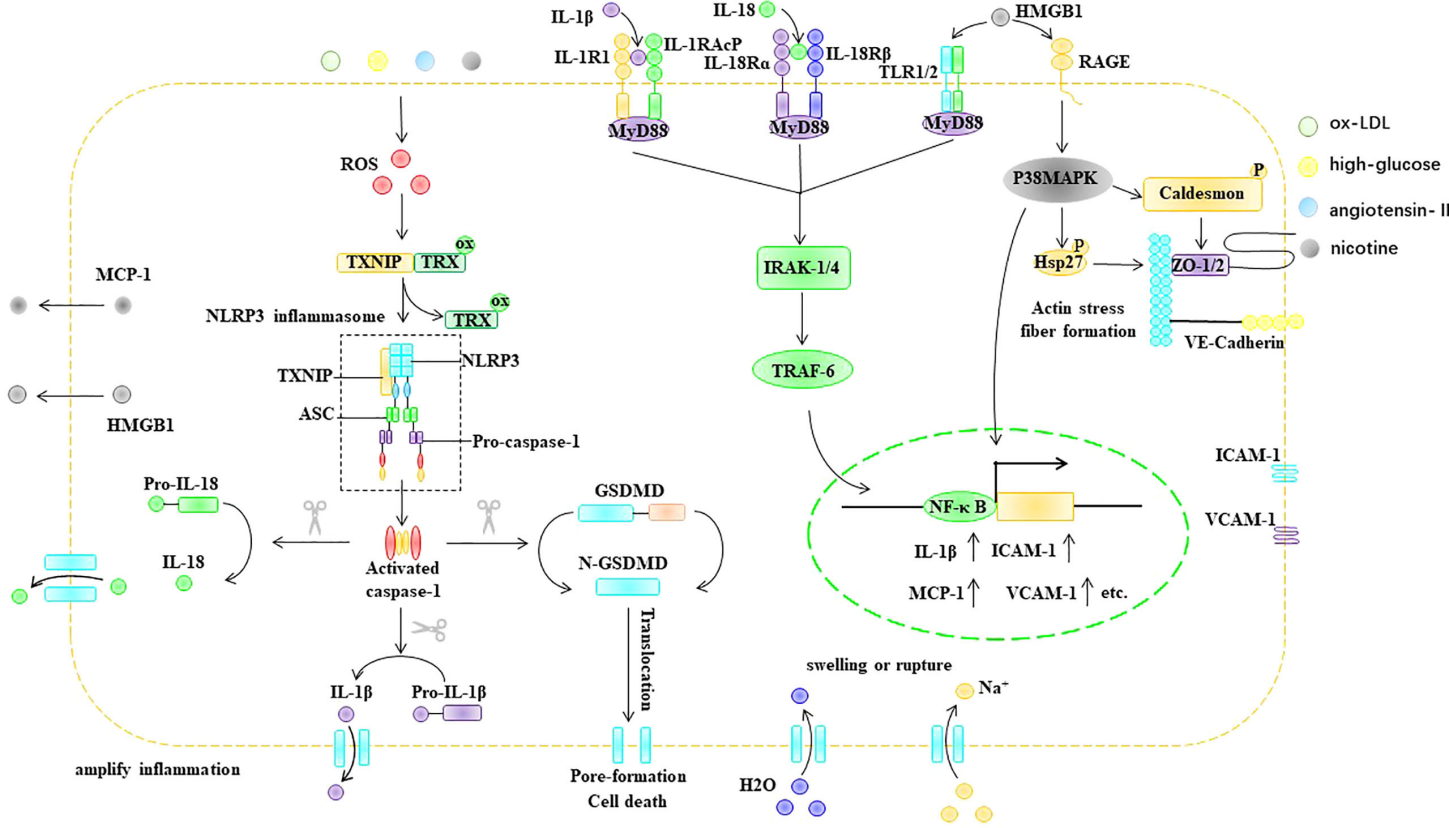
ROS trigger pyroptosis in EC. ASC, apoptosis-associated speck-like protein, ER endoplasmic reticulum, GSDMD Gasdermin D, HMGB1 high mobility group box 1, Hsp heat shock protein, ICAM-1 intercellular adhesion molecule-1, IL-1β interleukin-1β, IL-18 interleukin- 18, IL-1R IL-1 receptor, IL-18R IL-18 receptor, IRAK IL-1R-associated kinase, MCP-1 monocyte chemoattractant protein-1, MyD88 myeloid differentiation primary response gene 88, NF-κB nuclear factor kappa B, NLRP3 NLR-family pyrin domain-containing protein 3, ox-LDL oxidized low density lipoprotein, RAGE the receptor for advance glycation end products, ROS reactive oxygen species, TLR Toll-like receptors, Trx thioredoxin protein, TRAF TNF receptor-associated factor, TXNIP thioredoxin-interacting protein, VCAM-1 intervascular adhesion molecule-1.

In conclusion, stimulatory factors, such as ox-LDL, hyperglycemia, nicotine, and Ang II, can cause an increase in ROS in ECs. In endothelial cells, ROS acts as a bridge between pathological stimuli such as ox-LDL, hyperglycemia, Ang II, and nicotine and the activation of the NLRP3 inflammasome. The ROS-triggered NLRP3 inflammasome activation process in ECs involves two key steps: the initiation and activation phases. The initiation phase refers to the ROS-induced upregulation of NLRP3, caspase-1, IL-1β, and IL-18 in EC. The activation phase refers to ROS promoting the assembly and activation of the NLRP3 inflammasome through TXNIP. NLRP3 inflammasome activation promotes the maturation of IL-1β and IL-18 and can mediate the formation of porin N-GSDMD, thus causing cell swelling and even rupture, which lead to cell death. In addition, the formation of membrane pores promotes the release of cellular components, including IL-1β, IL-18, and HMGB1, which are involved in inflammatory responses. IL-1β, IL-18, and HMGB1 can bind to the EC surface at IL-1R, IL-18R, and TLR, respectively, and upregulate the expression of ICAM-1 and VCAM-1 *via* the Myd88/IRAK-1/TRAF-6/NF-κB pathway. In addition, NLRP3 inflammasome activation can disrupt tight junction proteins between EC, resulting in increased vascular permeability (**Figure 6**).

## 5 ROS trigger parthanatos in ECs

**Figure 7:**
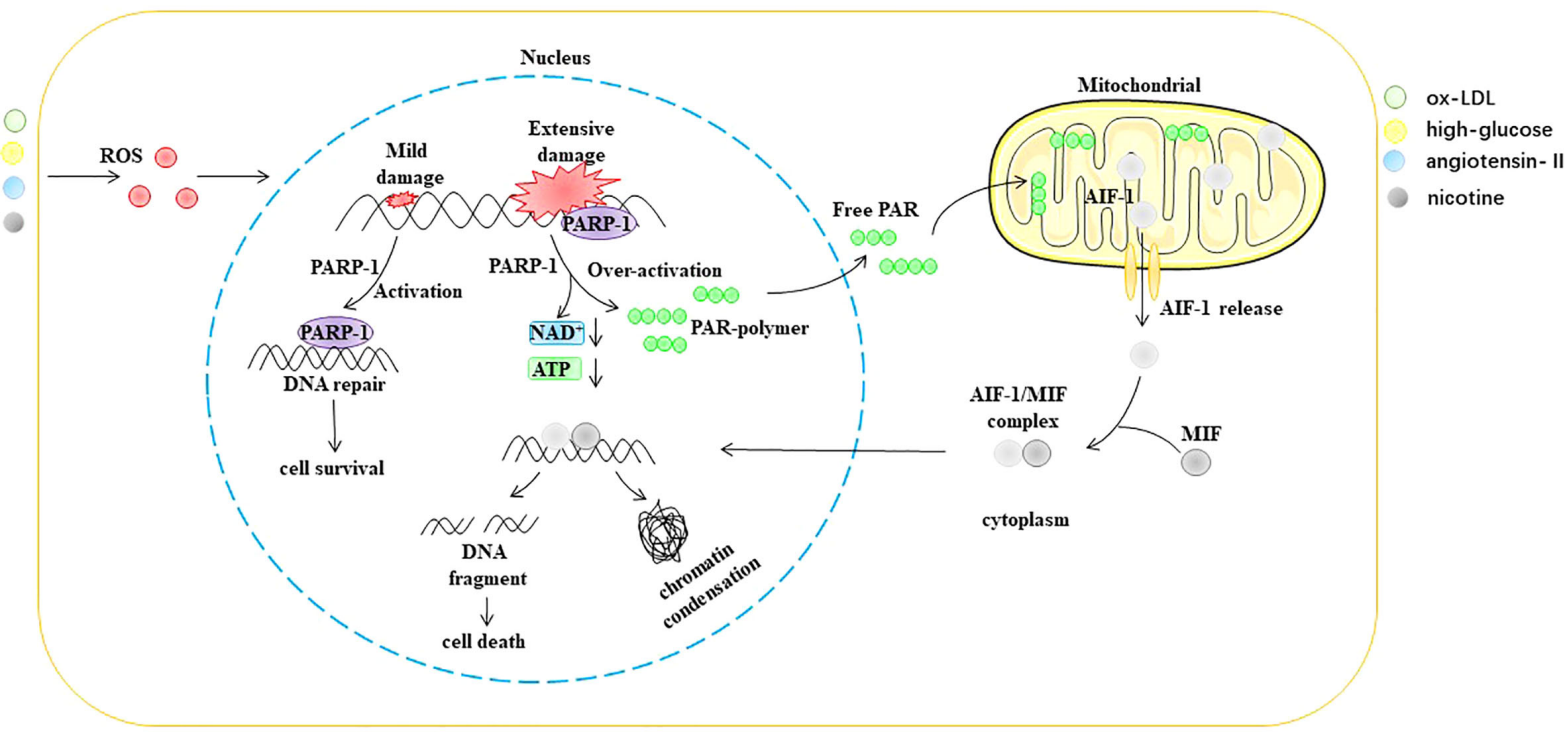
ROS trigger parthanatos in EC. AIF-1 apoptosis-inducing factor 1, ATP Adenosine triphosphate, MIF macrophage migration inhibitory factor, NAD+ nicotinamide adenine dinucleotide, ox-LDL oxidized low density lipoprotein, PAR poly (ADP-ribose), PARP-1 poly (ADP ribosome) polymerase 1, ROS reactive oxygen species.

Parthanatos is a type of programmed cell death that is dependent on poly (ADP-ribosome) polymerase 1 (PARP-1) (96, 97). PARP-1 is an ADP-ribosyltransferase that transfers ADP ribose from nicotinamide adenine dinucleotide (NAD+) to receptor proteins (98, 99). PARP-1 was originally described as a DNA nick sensor enzyme activated by DNA single- and double-strand breaks (100). DNA damage-induced activation of PARP-1 is considered the classical pathway for the activation of this enzyme. ROS, ionizing radiation, and alkylating agents are common causes of DNA fragmentation (101–103). PARP-1 activation depends on the degree of DNA damage. However, when DNA is extensively damaged, the overactivation of PARP-1 causes the accumulation of poly (ADP-ribose) (PAR), a process that consumes large amounts of NAD+. NAD+ is a direct substrate for the synthesis of PAR and a cofactor in many redox reactions, such as the tricarboxylic acid cycle, glycolysis, and pentose phosphate pathway (104, 105). Furthermore, the translocation of PAR from the nucleus to the mitochondria causes the release of apoptosis-inducing factor (AIF) (106, 107). After AIF leaves the mitochondria, it forms a complex with the macrophage migration inhibitory factor (MIF) in the cytoplasm. Subsequently, nuclear translocation of the AIF/MIF complex causes chromatin condensation and DNA fragmentation, ultimately leading to cell death (108–110) (**Figure 7**).

In recent years, many studies have shown that stimuli such as ROS, Ang II, ox-LDL, and hyperglycemia can trigger the occurrence of parthanatos in ECs. For example, Mathews et al. found that H_2_O_2_ and ONOO could activate PARP-1 in ECs, leading to EC death. Knockdown of PARP-1 inhibits H_2_O_2_ or ONOO^•^ triggered EC death (111). Liang et al. observed DNA damage and increased PARP-1 expression and activity in a model of Ang II-induced EC oxidative stress (112). Zhang et al. found that ox-LDL can induce coronary EC damage that is independent of caspase but dependent on the nuclear translocation of AIF (113). Wang et al. found that PARP1 was a key factor in the upregulation of arginase II (Arg II) induced by ox-LDL (114). Arg II results in reduced NO synthesis by competing with eNOS for the same substrate, l-arginine (115–117). PARP1 deficiency results in suppressed Arg II expression, enhanced eNOS expression, and improved NO production and endothelial function (114). Choi et al. found that enhanced PARP-1 activity is closely related to coronary artery endothelial dysfunction in mice with type 2 diabetes. The team further found that inhibition of PARP-1 activity restored eNOS phosphorylation and alleviated DNA damage, thereby improving ED (118). Taken together, ROS can cause extensive DNA damage that leads to the hyperactivation of PARP-1 and triggers parthanatos in EC.

## 6 ROS trigger ferroptosis in ECs

Ferroptosis is an iron-dependent process involving programmed cell death. Lipid peroxidation, which is the process by which OH^•^ attacks the carbon-carbon double bonds of lipids, particularly polyunsaturated fatty acids (PUFAs) (119), is an important marker for ferroptosis. Therefore, the production of OH^•^ is a key factor in the lipid peroxidation process. ${\text{O}}_{2}^{•–}$ reacts with H_2_O_2_ under the catalysis of Fe^2+^ to form Fe^3+^, OH^•^, and OH^-^, which is called the Fenton reaction. In addition, ${\text{O}}_{2}^{•–}$ reacts with Fe^3+^ to form Fe^2+^ in a process called the Haber-Weiss cycle. Lipid peroxidation can be divided into three stages: initiation, propagation, and termination. In the initial stage, OH^•^ interacts with lipids to form carbon-centred lipid radicals (L^•^). L^•^ reacts with oxygen to generate a lipid peroxy radical (LOO^•^), which further generates a new L^•^ (propagating phase) and lipid hydrogen peroxide (LOOH) from another molecular lipid. L^•^ and LOOH produced during the propagation stage can be terminated by antioxidant molecules of the mevalonate pathway, such as coenzyme Q10 (CoQ 10) and vitamin E (VitE) (120–122). In addition, studies have shown that iron chelators, such as deferoxamine (DFO) and ciclopirox olamine (CPX), can inhibit the occurrence of ferroptosis by inhibiting lipid peroxidation (122–125).

**Figure 8:**
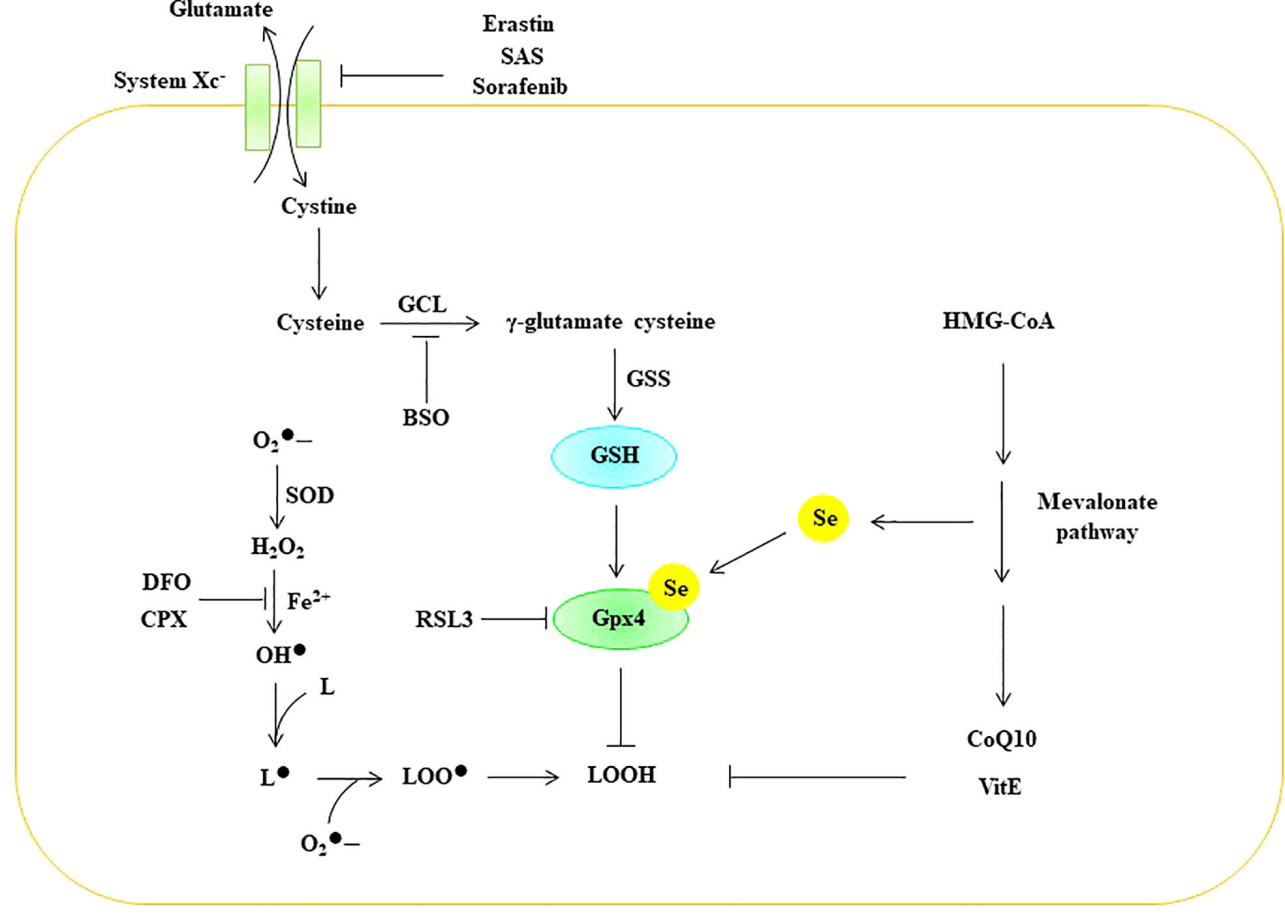
ROS trigger ferroptosis in EC. BSO Butionine sulfoxamine, CoQ 10 coenzyme Q10, CPX ciclopirox olamine, DFO deferoxamine GCL glutamate cysteine ligase, Gpx glutathione peroxidase, GSH glutathione, GSS GSH synthase, HMG-CoA 3-hydroxy-3-methyl-glutaryl-coenzyme A, Se selenium, SOD superoxide dismutase, SAS Sulfasalazine, VitE vitamin E, ${\text{O}}_{2}^{•-}$ superoxide, H_2_O_2_ hydrogen peroxide, OH^•^ peroxyl radical, L lipid, L^•^ lipid free radical, LOO^•^ lipid peroxy radical, LOOH lipid peroxide.

Lipid peroxidation is regulated by the glutathione antioxidant system, which is composed of glutathione (GSH), glutathione peroxidase (GPX), and glutaredoxin (GRX), and it can effectively prevent ROS overgeneration (126, 127). Glutamate, cystine, and glycine are the raw materials used for the synthesis of GSH. Cystine enters the cell through the amino acid antiporter Xc system, which is composed of the light chain subunit SLC7A11 and the heavy chain subunit SLC3A2. The Xc system exchanges cystine with glutamate in such a way that cystine enters the cell and is further reduced to cysteine (128). Glutamate cysteine ligase (GCL) catalyzes the formation of γ-glutamate-cysteine from glutamate and cysteine, which is the rate-limiting step in GSH synthesis. Subsequently, γ-glutamic acid cysteine and glycine are catalyzed by GSH synthase (GSS) to generate GSH (129). GSH effectively maintains Gpx in a reduced state. Gpx can effectively scavenge intracellular hydrogen peroxide and peroxide to maintain intracellular redox homeostasis (130). Eight different Gpxs (Gpx1-8) have been found in humans, and Gpx1-4 and Gpx6 are selenoproteins (131). Compared with other members of the Gpx family, Gpx4 is a lipid peroxidation-repair enzyme, and it can convert lipid peroxides (LOOH) to their corresponding alcohols (LOH) (122, 132). Therefore, Gpx4 is considered a central inhibitor of ferroptosis. Numerous studies have shown that inhibiting the Xc-GSH-Gpx4 antioxidant system can induce ferroptosis in cells. For example, erastin, sulfasalazine (SAS), and sorafenib initiate ferroptosis in cells by inhibiting the Xc system (133, 134). Butionine sulfoxamine (BSO) induces ferroptosis by inhibiting GCL (134), while RSL3 can initiate ferroptosis in cells by inhibiting the activity of Gpx4 (135) (**Figure 8**).

In recent years, studies have shown that ferroptosis is closely related to endothelial cell death. For example, Qin et al. found that zinc oxide nanoparticles (ZnONPs) could induce iron and lipid peroxidation in ECs in a dose- and time-dependent manner (136). The team further applied the lipid reactive oxygen species scavenger ferrostatin-1 and the iron chelator DFO to alleviate ZnONP-induced ferroptosis in ECs (22). Luo et al. showed that ferroptosis is related to ED and that the p53-xCT-GSH axis can regulate the process of EC ferroptosis (137). Sheng et al. showed that lysophosphatidylcholine (LPC) can induce increased intracellular iron and lipid peroxide levels and mitochondrial atrophy in EC. This process can be reversed by astragaloside IV (AS-IV) (138). Therefore, ferroptosis is an important mechanism by which ROS trigger programmed cell death in EC.

## 7 Outlook

In conclusion, hyperlipemia, hyperglycemia, nicotine and hypertension are common pathogenic factors causing impaired NO synthesis in EC, up-regulated expressions of pro-inflammatory cytokines and intercellular adhesion factors in EC, and EC death. ROS may be the common mechanism for these pathological activities (8, 9, 86, 91, 93, 95). Under pathological conditions, ROS in EC mainly originates from mitochondria, NOXs, eNOS uncoupling, and XO (4, 5). In addition, these pathways may independently or jointly cause excessive accumulation of ROS in EC. ROS can cause impaired NO synthesis through the eNOS uncoupling mechanism, thereby causing vasomotor dysfunction (7). ROS can up-regulate the expression of pro-inflammatory cytokines and intercellular adhesion factors in endothelial cells, such as IL-1β, IL-18, ICAM-1, VCAM-1, and E-selectin, which are participates the process of monocyte-endothelial cell adhesion, increased vascular permeability, and monocyte differentiation to macrophages (8, 9). Therefore, ROS is an important signal that mediates the involvement of EC in inflammatory responses. Furthermore, ROS can trigger endothelial cell death through different molecular mechanisms, including pyroptosis, parthanatos, and ferroptosis, which have been demonstrated in some animal models of disease. For example, Zhuang et al. found that Foxp1 expression was significantly downregulated in atherosclerosis-susceptible endothelium. The team further demonstrated that knockout of Foxp1 in ApoE-/- mice promoted the up-regulation of NLRP3, caspase-1 and Pro-IL-1β, increased IL-1β secretion, and enhanced monocyte adhesion, migration and Infiltrates into the vessel wall of the aortic root, thereby exacerbating the formation of atherosclerotic plaques (60). Wu et al. found that nicotine can mediate the pyroptosis of aortic endothelial cells and exacerbate the formation of atherosclerotic plaques by constructing an ApoE-/- mouse atherosclerosis model (93). Kong et al. found that targeting the P2X7/NLRP3 signaling pathway prevents retinal endothelial cell pyroptosis in diabetic retinopathy (139). Kasson et al. showed that enhanced NF-κB activity impairs vascular function in male type 2 diabetic mice through a PARP-1, Sp-1 and COX-2-dependent mechanism (140). Abdul et al. found that ferroptosis in brain microvascular endothelial cells of diabetic mice is closely related to vascular degeneration and neurovascular remodeling after stroke, and this process can be reversed by DFO (141). Therefore, how to effectively scavenge ROS may be an important target for the treatment of endothelial cell death-related diseases. For example, N-acetylcysteine ​​(NAC) is a potent ROS scavenger. Studies have shown that NAC inhibits pyroptosis, parthanatos, and ferroptosis by scavenging ROS (93, 142, 143). However, the following question about ROS-triggered EC death is unresolved and remains to be further explored: Do pyroptosis, parthanatos, and ferroptosis processes occur independently or simultaneously in the process of ROS-triggered EC death?

## Author contributions

KL, DZ and JL jointly completed the conception and writing of the review. HP, ZZ and RW completed the picture drawing of the review. All authors contributed to the article and approved the submitted version.

## Funding

This work was supported by the National Natural Science Foundation of China Grant 81970399.

## Conflict of interest

The authors declare that the research was conducted in the absence of any commercial or financial relationships that could be construed as a potential conflict of interest.

## Publisher’s note

All claims expressed in this article are solely those of the authors and do not necessarily represent those of their affiliated organizations, or those of the publisher, the editors and the reviewers. Any product that may be evaluated in this article, or claim that may be made by its manufacturer, is not guaranteed or endorsed by the publisher.

## Abbreviations

ASC apoptosis-associated speck-like protein; ALR AIM2-like receptors; Ang II angiotension; AIF apoptosis inducing factor; Arg II arginase II; ATP Adenosine triphosphate; BSO Butionine sulfoxamine; CARD caspase-recruitment domain; CoQ 10 coenzyme Q10; CPX ciclopirox olamine; DFO deferoxamine; DUOX dual oxidases**;** EC endothelial cell; ED endothelial dysfuction; ER endoplasmic reticulum; ET-1 endothelin-1; ETC electron transport chain; FAD flavin adenine dinucleotide; FIIND function-to-find domain; Forkhead box P transcription factor 1 Foxp1; GSDMD Gasdermin D; Gpx glutathione peroxidase; Grx glutaredoxin; GSH glutathione; GSS GSH synthase; HMG-CoA 3-hydroxy-3-methyl-glutaryl-coenzyme A; HMGB1 high mobility group box 1; Hsp heat shock protein; ICAM-1 intercellular adhesion molecule-1; IL-1β interleukin-1β; IL-18 interleukin-18; IL-1R IL-1 receptor; IL-18R IL-18 receptor; IRAK IL-1R-associated kinase; LDH lactate dehydrogenase; LPS lysophosphatidylcholine; MIF macrophage migration inhibitory factor; Mo-co molybdenum cofactor; MyD88 myeloid differentiation primary response gene 88; NAC N-acetylcysteine; NAD+ nicotinamide adenine dinucleotide; NBD nucleotide-binding domain; NLRs NOD-like receptor proteins; NLRP3 NLR-family pyrin domain-containing protein 3; NNT nicotinamide nucleotide transhydrogenase; NO nitric oxide; NOX NAPDH oxidase; NOS nitric oxide synthase; ox-LDL oxidized low density lipoprotein; OXPHO oxidative phosphorylation; PUFAs polyunsaturated fatty acids; PAR poly (ADP-ribose); PARP-1 poly (ADP-ribosome) polymerase 1; PFD pore-forming domain; PKC protein kinase C; PYD pyrin domain; Poldip2 polymerase delta-interacting protein 2; RD repressor domain; ROS reactive species oxygen; SOD superoxide dismutase; SAS Sulfasalazine; Se selenium; TRAF TNF receptor-associated factor; Trx reduced thioredoxin protein; TXNIP thioredoxin-interacting protein; t-BHP tert-butyl hydroperoxide; XO xanthine oxidase; XDH xanthine dehydrogenase; XOR xanthine oxidoreductase; VCAM-1 intervascular adhesion molecule-1; VitE vitamin E; ZnONPs zinc oxide nanoparticles

## Glossary

**Table:**

ASC	apoptosis-associated speck-like protein
ALR	AIM2-like receptors
Ang II	angiotension
AIF	apoptosis inducing factor
Arg II	arginase II
ATP	Adenosine triphosphate
BSO	Butionine sulfoxamine
CARD	caspase-recruitment domain
CoQ 10	coenzyme Q10
CPX	ciclopirox olamine
DFO	deferoxamine
DUOX	dual oxidases
EC	endothelial cell
ED	endothelial dysfuction
ER	endoplasmic reticulum
ET-1	endothelin-1
ETC	electron transport chain
FAD	flavin adenine dinucleotide
FIIND	function-to-find domain
Foxp1	Forkhead box P transcription factor 1
GSDMD	Gasdermin D
Gpx	glutathione peroxidase
Grx	glutaredoxin
GSH	glutathione
GSS	GSH synthase
HMGCoA	3-hydroxy-3-methyl-glutaryl-coenzyme A
HMGB1	high mobility group box 1
Hsp	heat shock protein
ICAM-1	intercellular adhesion molecule-1
IL-1b	interleukin-1b
IL-18	interleukin-18
IL-1R	IL-1 receptor
IL-18R	IL-18 receptor
IRAK	IL-1R-associated kinase
LDH	lactate dehydrogenase
LPS	lysophosphatidylcholine
MIF	macrophage migration inhibitory factor
Mo-co	molybdenum cofactor
